# Inhibition of Lung Tumor Development in ApoE Knockout Mice via Enhancement of TREM-1 Dependent NK Cell Cytotoxicity

**DOI:** 10.3389/fimmu.2019.01379

**Published:** 2019-06-18

**Authors:** Yong Sun Lee, In Jun Yeo, Ki Cheon Kim, Sang-Bae Han, Jin Tae Hong

**Affiliations:** College of Pharmacy and Medical Research Center, Chungbuk National University, Cheongju, South Korea

**Keywords:** lung tumor development, apolipoprotein E, TREM-1, T-bet, NK cells

## Abstract

Apolipoprotein E (ApoE) is known to regulate lipid homeostasis and associated with atherosclerogenesis. Eventhough atherosclerogenesis is associated with tumor development, the role of ApoE in lung tumorigenesis and metastasis is not clear. Thus, the tumor growth and metastasis were compared in WT and ApoE knockout (KO) mice. Urethane-induced lung tumor incidence and B16F10 lung metastasis in ApoE knockout (KO) mice were significantly reduced in comparison to that in WT mice. Knockdown of ApoE expression in lung cancer cells and B16F10 cells also decreased cancer cell growth and metastasis. The inhibitory effect of ApoE KO on tumor development and metastasis was associated with increase of infiltration of NK cells. NK cells derived from ApoE KO mice showed much greater cytotoxicity than those from WT mice. These cytotoxic effect of NK cells derived from ApoE KO mice was associated with higher expression of Granzyme B, Fas Ligand, IFN-γ, TNF-α, NKG2D, NKp46, and DNAM-1 expression. Triggering receptor expressed on myeloid cell (TREM)-1 is a proinflammatory mediator expressed on NK cells, and is known to be associated with NK cell cytotoxicity. Thus, we investigated the role of TREM-1 on ApoE KO mice originated NK cell mediated cytotoxicity for cancer cells. Blockade of TREM-1 expression with a TREM-1 antagonist prevented NK cell-mediated cytotoxicity. TREM-1 antibody recovered cytotoxic effect of NK cells derived from KO mice of T-bet, which upregulating gene for TREM-1. These data indicate that ApoE KO suppressed lung tumor development and metastasis via increase of TREM-1-dependent anti-tumor activity of NK cells.

## Introduction

As one of the most common malignancies, lung cancer is a leading cause of death, and is expected to cause increased future mortality (1). The majority of lung cancers originate from epithelial cells and can be divided into non-small cell lung cancer (NSCLC) and small cell lung cancer (SCLC), morphologically. NSCLC is predominant type of lung cancer and accounts for about 85% of lung cancers (2). Although smoking is the primary cause of most lung cancer cases, approximately 10–15% are caused by genetics and other factors including nutritional factors (3). Among the nutritional factors, dietary cholesterol is associated with an increased risk of lung cancer (4).

Cholesterol serves as a metabolic precursor to other bioactive sterols and plays a major role in the structure of the plasma membrane (5, 6). Previous studies have demonstrated that an elevated level of cholesterol in the plasma membrane may alter membrane-associated signaling proteins such as receptor tyrosine kinases (RTKs), reduce apoptosis, and increase tumor growth (7). The results of numerous epidemiological studies also suggest that dysregulated cholesterol metabolism might be a key factor linking dyslipidemia and tumor development (8, 9). Cholesterol has been reported to be associated with NSCLC development in patients (10). In addition, it was reported that occurrence of certain malignancies is reduced in patients consuming 3-hydroxy-3-methyl-glutaryl CoA (HMG-CoA) reductase inhibitors (statins), used for the treatment of dyslipidemia (8, 11). ApoE, one of the apolipoprotein isoforms, regulates lipid homeostasis via cholesterol transport and metabolism (12). Even though the significant role of ApoE in cholesterol homeostasis and the significance of cholesterol in tumor growth are well-known, the role and mechanism of ApoE in tumor cell growth are not clear.

ApoE itself has been shown to act as an autocrine or paracrine growth factor to influence carcinogenesis (13). In human lung cancer, the levels of ApoE gene expression were significantly higher in cancer tissue than in adjacent non-cancer tissue (14). Serum ApoE is associated with lymph node metastasis in lung adenocarcinoma patients (15). It was also reported that high expression of ApoE promotes cancer proliferation and migration and contributes to an aggressive clinical course in patients with lung adenocarcinoma (16). In ovarian cancer, suppression of ApoE decreased ApoE-induced cancer cell proliferation and survival (13). Moreover, ApoE overexpression contributes to cisplatin resistance and can serve as a poor prognostic biomarker for patients with lung adenocarcinoma (16). The levels of ApoE are significantly elevated in malignant pleural effusions of lung cancer patients (17). However, it was also reported that ApoE is involved in the inhibition of melanoma metastasis and angiogenesis (18). Recent studies revealed that ApoE KO mice showed much greater orthotopic mammary tumor development and pulmonary metastases than WT mice (19). These conflicting evidences indicate that the precise role of ApoE on tumor development could be affected by tumor environment factors.

Natural killer (NK) cells and cytotoxic T (Tc) cells are major immune cells that have a critical role in host defense against pathogens and transformed cells (20, 21). The relationship between ApoE and the above cells has been studied mainly in atherosclerosis. CD8+ Tc cells and NK cells infiltrated atherosclerotic lesions and led to development of atherosclerotic plagues via apoptosis of macrophages, smooth muscle cells, and endothelial cells in ApoE KO mice (22). It was also reported that the lack of ApoE caused increased proliferation of T cells and elevated secretion of IFN-γ *in vitro*, and increased expression of MHC class II and co-stimulatory surface proteins CD40 and CD80, crucial for T cell activation, in monocytes and macrophages (23). However, the specific role of ApoE related to anti-tumor activity of NK cells and Tc cells is not clear.

The TREM (triggering receptor expressed on myeloid cells) family has been known to play an important role in innate immune responses (24). TREM-1 is a pro-inflammatory mediator expressed on NK cells, monocytes, neutrophils, granulocytes, and dendritic cells, and is usually upregulated due to infections of bacteria and fungi, and sepsis (24, 25). It has been reported that TREM-1 promoted atherosclerosis via monopoiesis, pro-inflammatory cytokine release, and foam cell formation in ApoE KO mice (26). Suppression of TREM-1 reduced CD4+ T cell activation, and proliferation of IFN-γ-producing Th1 cells in a cardiac allograft model (27). Recent studies showed that TREM-1 is also associated with lung cancer and hepatocellular carcinoma development. Cancer cells can directly upregulate TREM-1 expression in macrophages in lung cancer patients, and this is linked with cancer recurrence and poor survival of non-small cell lung cancer patients (28). However, blockade of TREM-1 suppressed tumor growth in a human lung cancer xenograft model (29). In hepatocellular carcinoma, TREM-1 deletion decreased diethylnitrosamine-induced liver cancer by attenuating Kupffer cell activation (30). Thus, it is possible that TREM-1 and TREM-1-mediated cytotoxic effects of NK cells could be associated with carcinogenesis in ApoE knockout mice.

Therefore, in this study, we investigated the effect of ApoE KO modulation of lung tumor development and metastasis, and the significant of TREM-1 and NK cells in urethane-induced lung tumor development and metastasis in ApoE knockout mice.

## Materials and Methods

### Cell Culture

A549 and NCI-H460 human lung cancer cells and B16F10 mouse melanoma cells were obtained from the American Type Culture Collection. A549, NCI-H460 or B16F10 cells were grown in Roswell Park Memorial Institute 1640 (RPMI 1640) or Dulbecco Modified Eagle Medium (DMEM) supplemented with 10% fetal bovine serum (FBS), penicillin (100 units/ml) and streptomycin (100 μg/ml) at 37°C in a humidified atmosphere of 5% CO_2_ air. In cholesterol treatment, cholesterol (Sigmal-Aldrich) was dissolved in distilled water and cholesterol solution was added <1% to assay medium. Mouse splenic NK cells from WT mice, ApoE KO mice and T-bet KO mice were isolated by negative selection using a mouse NK isolation kit (Purity around 90%; Miltenyi Biotec). Isolated NK cells were cultured in RPMI 1640 supplemented with 10% FBS, penicillin (100 units/ml) and streptomycin (100 μg/ml), 50 μM 2-mercaptoethanol and recombinant human IL-2 (3,000 units/ml, Bayer HealthCare Pharmaceuticals). IL-2-expanded NK cells were used from day 8–12.

### Transfection

Lung cancer cells and B16F10 cells were transfected with negative control (NC) or ApoE siRNA (Santa Cruz Bio) using Lipofectamine® RNAiMAX reagent (Invitrogen) in Opti-MEM, following the manufacturer's protocol. For ApoE stable knockdown cells, B16F10 cells were transfected with NC or ApoE shRNA (pGFP-V-RS vector; Origene) using Lipofectamine® 3000. Transfected cells were cultured with Puromycin (Sigma-Aldrich) for 3 weeks. Puromycin-resistant colonies were selected and expanded.

### Animals

The genetic background of ApoE KO and T-bet KO mice is C57BL/6. The ApoE KO mice were purchased from the Jackson laboratory (Bar Harbor, ME, USA), T-bet KO mice were kindly provided from Dr. Eun Sook Hwang (Ewha Womans University, Seoul, Korea) and C57BL/6 WT mice were obtained from DBL (Eumsung, Korea). The mice were kept at the Laboratory Animal Research Center in Chungbuk National University, Korea. The mice were housed under specific pathogen-free conditions at 22 ± 1°C, a relative humidity of 55 ± 10% and a 12 h of light per day. The mice were given access to solid diets and sterilized water *ad libitum*. All animal experiments were approved and carried out according to the Guide for the Care and Use of Animals [Chungbuk National University Animal Care Committee, Korea (CBNUA-929-16-01)].

### Carcinogenesis Protocol

Eight-week-old WT and ApoE KO mice were used for carcinogenesis. Mice were fed with normal diet and intraperitoneally injected with 1 mg/g urethane (ethyl carbamate; Sigma-Aldrich) once a week for 10 weeks and sacrificed after 6 months. After sacrifice, lungs were fixed in 4% formalin solution. Then, lung tumors were counted and embedded in paraffin.

### Lung Metastasis Protocol

Eight-week-old WT and ApoE **KO** mice were used for lung metastasis. Mice were fed with normal diet or high-fat diet from 1 week before injection until sacrifice. Mice were intravenously injected at tail-vein with B16F10 cells (4 × 10^4^ cells/mouse). In lung metastasis using ApoE stable knockdown cells, WT mice were fed with normal diet and intravenously injected at tail-vein with NC or ApoE stable knockdown B16F10 cells (4 × 10^4^ cells/mouse). After 3 weeks, all mice were sacrificed. Then B16F10 lung metastatic nodules on lung surface were counted and fixed in 4% formalin solution. Then, lungs were embedded in paraffin.

### Cytotoxic Assay

NK cell–mediated cancer cell growth was measured by a lactate dehydrogenase (LDH) detection assay kit (TaKaRa Bio Inc.) according to the manufacturer's protocol. The target cells, B16F10 mouse melanoma, were mixed with effector cells, splenic NK cells from WT mice or ApoE KO mice, in U-bottomed 96-well plate at the different NK cell/cancer cell ratios. In agonist and antagonist experiment, WT NK cells were pretreated with LP-17 peptide (antagonist, 100 ng/ml, Peptron) or anti-TREM-1 mAb (agonist, 10 μg/ml, MAB1187; R&D systems) for 24 h. Then co-cultured with B16F10 cells as target cells for 4 h, the supernatant was collected by centrifugation and transferred to flat-bottomed 96-well plate. Supernatant was analyzed by LDH release (yellow tetrazolium as substrate). Samples were measured using a spectrophotometric microplate reader at 490 nm. The percentage of specific lysis was calculated as follows: (experimental release – target spontaneous release – effector spontaneous release)/(target maximum release – target spontaneous release) × 100% (31).

### MTT Assay

Cell proliferation was measured by performing a thiazolyl blue tetrazolium bromide (MTT) assay to the detect living cells. A MTT solution (5 mg/ml v/v, Sigma-Aldrich) in phosphate-buffered saline (PBS) was directly added to the wells and then incubated for 2 h to allow MTT to metabolize to formazan. After incubation, the MTT contained medium was aspirated and dimethyl sulfoxide was added. Absorbance was measured with a spectrophotometric plate reader at 570 nm. The data were normalized to their respective controls and are presented as a bar graph.

### Western Blot Analysis

Lung tissues, A549, NCI-H460 and NK cells were lysed by Pro-prep protein extraction buffer (iNtRON) and the total protein concentration was determined using the Bradford reagent (Bio-Rad). Equal amounts of lysates were transferred to Immobilon® PVDF membranes (Millipore) and the membranes were immunoblotted with specific primary antibodies. The membranes were washed and incubated with diluted horse radish peroxidase-conjugated secondary antibodies. After washes, the membranes were detected using an enhanced chemiluminescence kit (Millipore). The band intensities were measured using the Fusion FX 7 image acquisition system (Vilber Lourmat, Eberhardzell, Germany). Primary antibody information was described in Supplementary Table 1.

### Immunohistochemistry

Human lung cancer tissue microarrays were purchased from US Biomax (LC1503). Paraffin-embedded human and mouse tumor tissue sections were blocked for 60 min with 2% normal horse or goat serum contained blocking solution diluted in 1X PBS. To prevent non-specific binding, tissues sections were blocked using Avidin/Biotin-blocking solution (Thermo Fisher Scientific). The sections were incubated with specific primary antibodies in blocking solution for overnight at 4°C. And then, the sections were washed three times for 10 min each in PBS and incubated in biotinylated anti-mouse, rabbit and goat antibody for 90 min. The sections were washed three times for 10 min each in PBS, followed by formation of the avidin/biotin-peroxidase complex (Vector Laboratories). The slides were washed, and the peroxidase reaction developed with diaminobenzidine and peroxide and then counter-stained with hematoxylin, mounted in Cytoseal XYL (Thermo Fisher Scientific) and evaluated on a light microscope (x100 or x200, Olympus, Tokyo, Japan). Tissue microarray images were analyzed by Image J software and IHC Profiler plugin (32). Immunohistochemical images were scored automatically and scored values are Negative, Low positive, Positive and High positive. Primary antibody information was described in Supplementary Table 1.

### Real-Time Quantitative PCR

Total RNA from NK cells from WT mice and ApoE KO mice was extracted by easy-BLUE Total RNA Extraction Kit (iNtRON) and cDNA was synthesized using High Capacity RNA-to-cDNA kit (Applied Biosystems). Quantitative real-time PCR was performed using custom-designed primers and 18s was used for house-keeping control in a StepOnePlus™ Real-Time PCR System (Applied Biosystems, Foster City, CA, USA) (Supplementary Table 2). Thermocycling conditions consisted of an initial denaturation of 2 min at 95.0°C, followed by 40 cycles of 95.0°C for 5 s and 60.0°C for 10 s. The values obtained for the target gene expression were normalized to 18s and quantified relative to the expression in control samples.

### Wound Migration Assay

Wound migration assay using μ-Slide was performed according to the manufacturer's recommendations (Ibidi). Briefly, B16F10 cells were seeded on μ-Slide at 1 × 10^4^ cells/well. After overnight culture, cells were transfected for 24 h. Then, silicon wells were removed, and growth medium changed to cell free medium. Images were captured by a phase microscopy (x40, Nikon, Tokyo, Japan) at every hour until gap closed.

### Transwell Migration Assay

Serum-starved B16F10 cells (1 × 10^5^ cells in 100 μl serum-free medium) were added to the collagen pre-coated transwell insert (pore size 8.0 μm; distance 6.5 mm), and the culture well was filled with 600 μl of the complete medium containing 10% FBS. After 18 h, cells were fixed with 70% ethanol for 10 min and stained with crystal violet solution. Then, non-migrated cells in the upper side of transwell insert were removed using a cotton swab. Three random fields of each insert were counted and photographed under a light microscope (x100, Olympus, Tokyo, Japan).

### Patient Data Analysis

ApoE expression in lung cancer patients, patient survival and recurrence was analyzed through Okayama database using Oncomine (www.oncomine.org) (33).

### Gene Network Analysis

The gene network between ApoE, TREM-1 and T-bet was analyzed using GeneMANIA (www.genemania.org) (gene-gene interactions based on attributions: co-expression, co-localization, genetic interactions, pathway, physical interactions, predicted interactions and shared protein domains).

### Statistics

Statistical analyses of the data were evaluated using Graphpad Prism Software (San Diego, CA, USA). Pairwise comparisons were performed using 1-way ANOVA test and the differences were evaluated by Student's *t*-tests. The data are presented as mean ± S.E.M. A value of *p* < 0.05 was considered statistically significant.

## Results

### Inhibition of Lung Tumor Development and Metastasis in ApoE KO Mice

**Figure 1:**
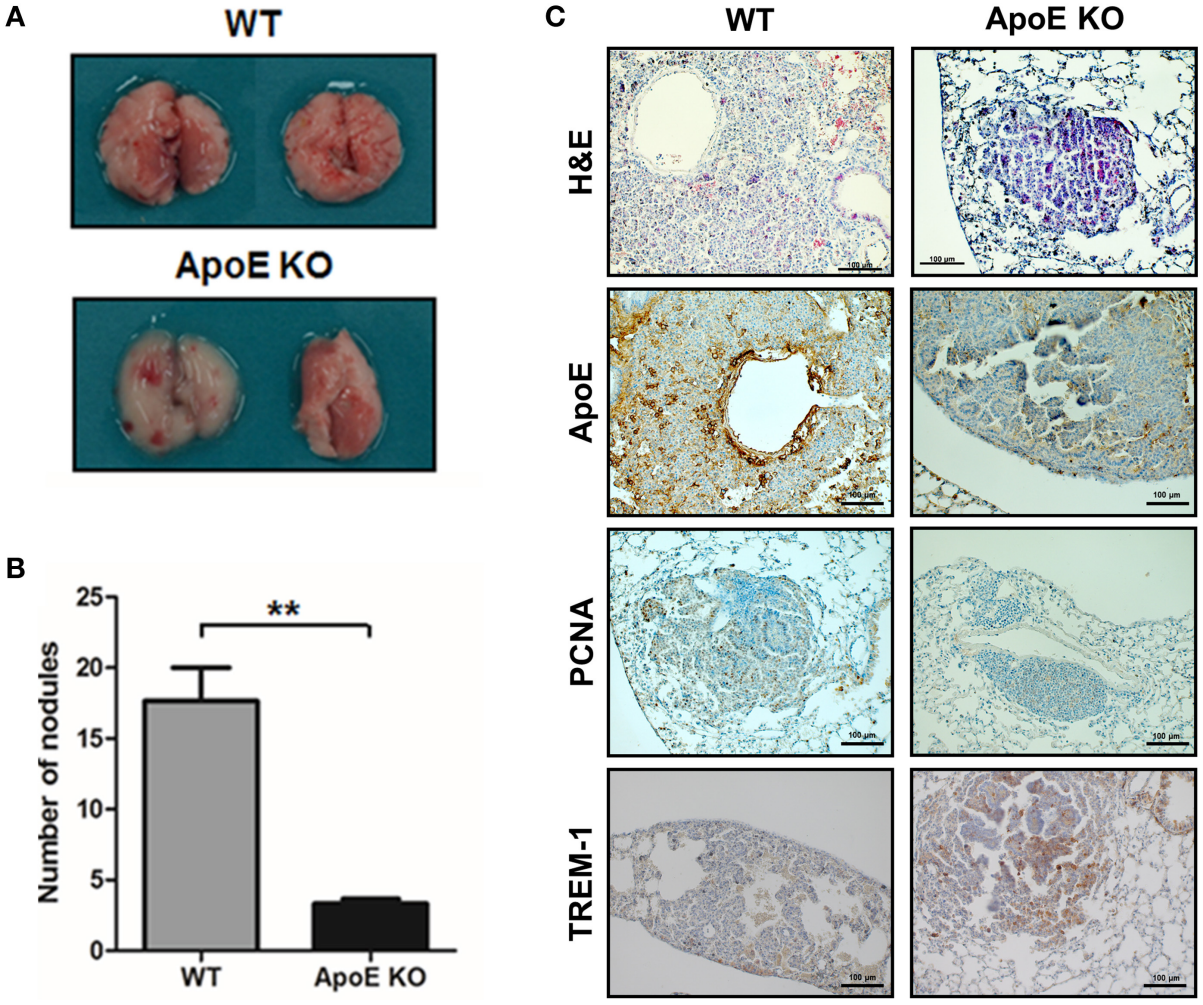
Effect of ApoE knockout on the lung tumor development. **(A,B)** Tumors were induced by a single intraperitoneal injection of 1 mg/g urethane once a week for 10 weeks (*n* = 6). Mice were sacrificed at 6 months after injection of the carcinogen. At the time of sacrifice, numbers of urethane-induced lung tumor were counted. **(C)** Lung tissues were processed and stained with H&E or analyzed by immunohistochemistry for detection of positive cells for ApoE, PCNA, and TREM-1. Scale bar, 100 μm. ^**^p < 0.01.

**Figure 2:**
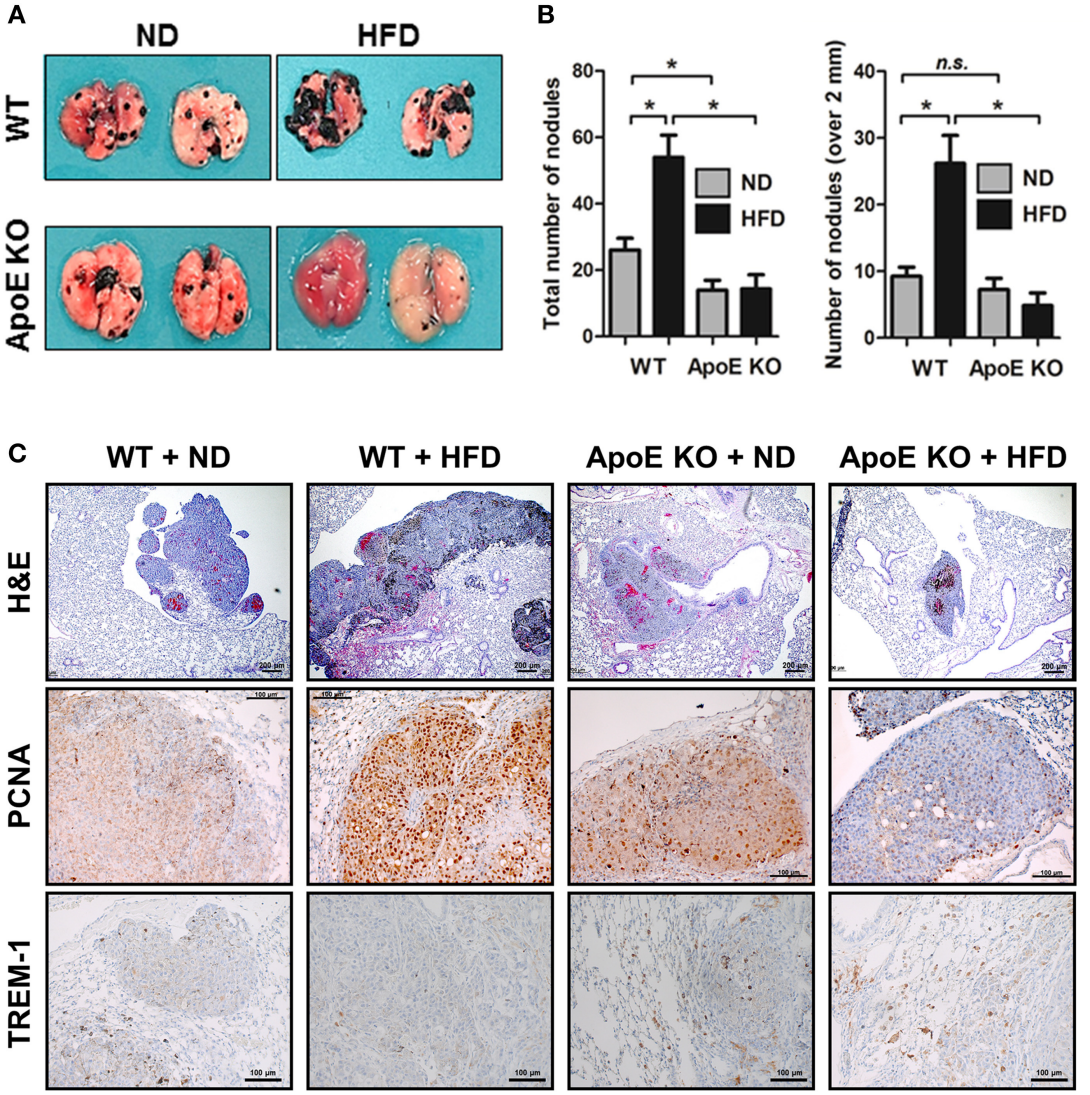
Effect of ApoE knockout on B16F10 lung metastasis. **(A,B)** B16F10 cells were intravenously injected at mouse tail vein (4 × 10^4^ cells/mouse) (*n* = 6). After 21 days, mice were sacrificed, and lung metastatic nodules were visualized and counted. **(C)** Lung metastasis tissues were stained with haematoxylin and eosin or analyzed by immunohistochemistry for detection of positive cells for PCNA and TREM-1. Scale bar, 100 μm. ^*^p < 0.05.

In this study, we investigated the role of ApoE in lung tumor development and metastasis using ApoE KO mice. We found that carcinogen-induced (urethane 1 mg/g) lung tumors in hypercholesterolemic ApoE KO mice were smaller than those in WT mice (Figures 1A,B). The average number of adenomas was 17.6 ± 4.0 in WT mice, but only 3.3 ± 0.5 in ApoE KO mice. The histological analysis showed that the tumor size in ApoE KO mice were significantly smaller compared than WT mice. PCNA, a proliferation marker, positive cell number was lower in ApoE KO mice than WT mice (Figure 1C). Circulating tumor cells efficiently colonize into lung tissue due to its large surface area and rich blood supply (34). In addition, high cholesterol affects cancer metastasis and growth (19). We intravenously injected an identical number of melanoma cells (B16F10) into ApoE KO and WT mice fed with normal diet (ND) or high-fat diet (HFD) and quantified metastatic lesions in the lungs after 3 weeks. We found significantly less metastasis in ApoE KO mice than in WT mice. The number of metastatic nodules were 26.1 ± 14.3 in WT mice and 14.0 ± 5.9 in ApoE KO mice (Figures 2A,B). More metastases were seen in HFD-fed WT mice (54.0 ± 23.0) than in ND-fed WT mice (26.1 ± 14.3), but there was no difference in metastases between HFD-fed (14.3 ± 10.5) and ND-fed ApoE KO mice (14.0 ± 5.9). Histological analysis showed lung metastatic tumors were less-differentiated in ApoE KO mice (Figure 2C). The number of PCNA positive cells in lung metastatic tumors was lower in ApoE KO mice compared with WT mice (Figure 2C). To investigate whether cholesterol itself could affect on cancer cell growth, we determined cancer cell growth after treatment of cholesterol. However, cholesterol did not affect cell proliferation in lung cancer cells (A549 and NCI-H460) and B16F10 mouse melanoma cells (Supplementary Figure 1). These data suggest that the inhibition of tumor growth and metastasis in ApoE KO mice may not be related to the cholesterol level itself, but could be associated with the physiological effects of ApoE.

### Changes of the Expression of Cell Cycle Regulatory, Metastasis-Related, and Apoptosis-Related Proteins in Tumor Tissues of ApoE KO Mice

Following the *in vivo* studies, we investigated the role of ApoE in tumor development-related signaling pathways associated with cell cycle, metastasis, and apoptosis. Western blot data showed that the protein levels of PCNA, CDK4, CDK6, Cyclin D1, MMP-2, MMP-9, and Bcl-2 were decreased, but cleaved caspase-3 was increased in tumor tissues of ApoE KO mice compared to those of WT mice (Supplementary Figure 2A). The phosphorylation of p38 and STAT3 was also decreased in tumor tissues of ApoE KO mice compared to those of WT mice (Supplementary Figure 2A). Next, we measured metastasis-related protein expression in lung metastatic tumor tissues. Western blot analysis revealed that MMP-2 and MMP-9 were lower in ApoE KO mice than in WT mice (Supplementary Figure 2B). We also found that expression of these cell growth, metastasis-related and anti-apoptosis-related proteins was decreased, but the expression of apoptotic protein was increased in HFD-fed ApoE KO mice (Supplementary Figure 2B).

### ApoE-Knockdown Inhibits Cell Proliferation and Migration in Cancer Cells

**Figure 3:**
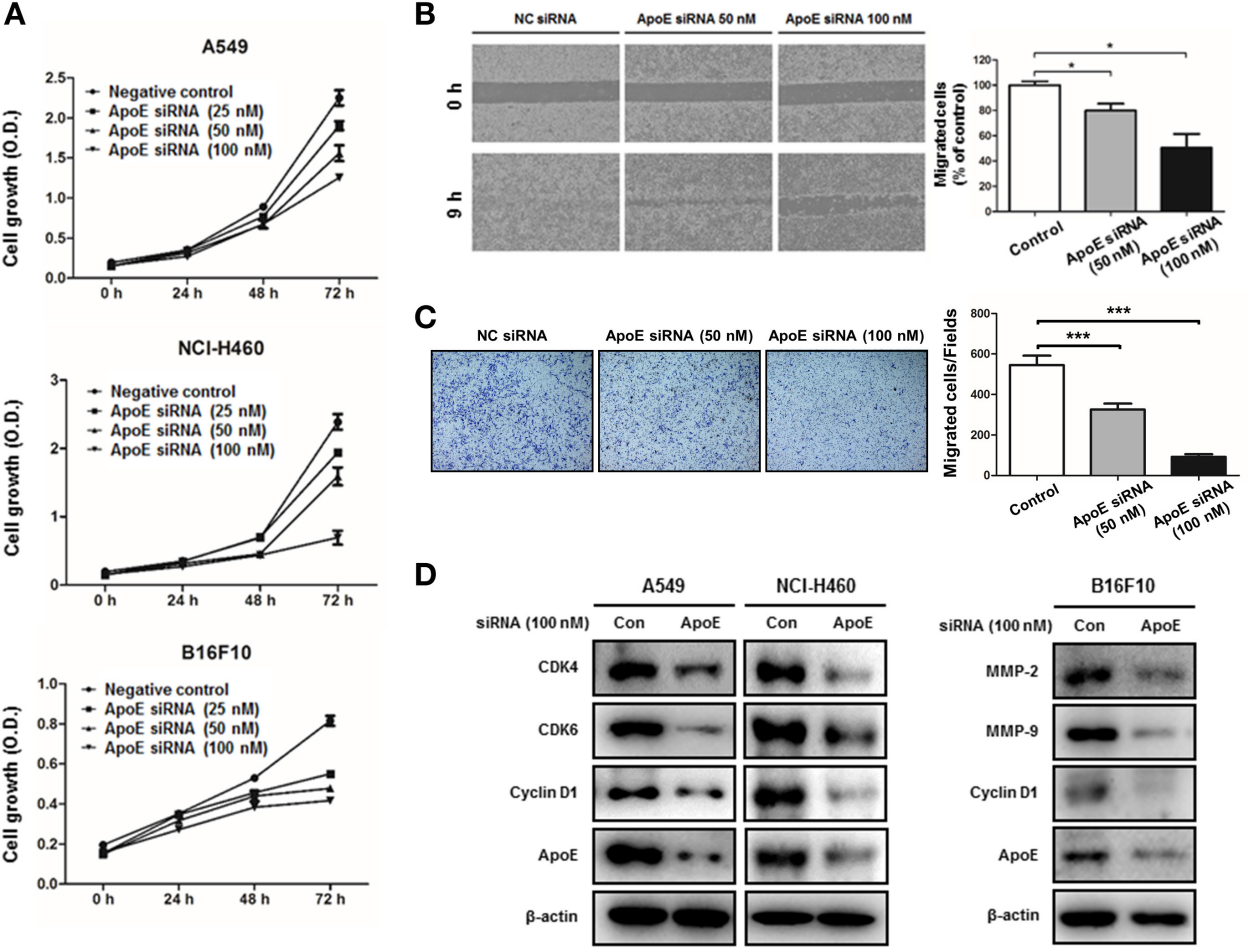
Effect of ApoE knockdown on cell growth and migration in cancer cells. **(A)** Lung cancer cells (A549 and NCI-H460) and B16F10 cells were plated on 96-well plates (1 × 10^3^ cells per well) and transfected with the negative control (NC) siRNA or ApoE siRNA (25, 50 or 100 nM) for indicated time points. Cell growth was measured by MTT assay (*n* = 5). **(B)** B16F10 cells were seeded on μ-Slide and transfected with NC siRNA or ApoE siRNA (50 or 100 nM). Cells were cultured to confluent on μ-Slide (*n* = 3). After silicon-wall removal, cells were allowed to migrate into cell-free zone. Cell migration was detected at various times post-silicon-wall removal by a microscopy at 100X. **(C)** B16F10 cells transfected with NC siRNA or ApoE siRNA (50 or 100 nM) were seeded onto transwell inserts pre-coated with collagen on the bottom side and loaded into culture well filled with growth medium containing 10% FBS as a chemoattractant (*n* = 3). After 18 h incubation, transwell inserts were fixed and stained by crystal violet solution. Bar graphs represent cell-migration distance or number of migrated cells. ^*^p < 0.05 and ^***^p < 0.01. **(D)** Cells were transfected with NC siRNA or ApoE siRNA (100 nM) for 24 h. Cell extracts were analyzed by Western blotting. Samples (30 μg) were resolved on SDS–PAGE and detected with specific antibodies against CDK4, CDK6, Cyclin D1, MMP-2, MMP-9 and ApoE. β-actin was used as a loading control.

To investigate the role of ApoE *in vitro*, we suppressed ApoE expression using siRNA. Inhibition of ApoE expression resulted in the inhibition of lung cancer cell (A549 and NCI-H460) proliferation in a time-dependent manner compared to cells transfected with negative control siRNA (Figure 3A). However, these inhibitory effects were not altered in the presence of cholesterol (Supplementary Figure 1). Using wound scratch assay and trans-well migration assay, we determined whether ApoE expression affects B16F10 melanoma migration. ApoE knockdown cells showed reduced migration compared to control cells in the wound scratch assay (Figure 3B). In the trans-well migration assay, ApoE knockdown cells also showed lesser migration than control cells (Figure 3C). Expression of cell cycle regulatory proteins CDK4, CDK6, and cyclin D1 was significantly decreased in ApoE knockdown lung cancer cells (Figure 3D). In B16F10 cells, expression of MMP-2, MMP-9, and cyclin D1 was decreased when ApoE knockdown cells (Figure 3D). Phosphorylation of p38 and STAT3 was also decreased in ApoE knockdown cells (Supplementary Figure 3). In addition, we conducted lung metastasis using NC and ApoE stable knockdown (ApoE-shRNA) cells (Supplementary Figure 4A). We intravenously injected NC and ApoE-shRNA cells into tail-vein of mice. We found that lung metastatic nodules were significantly reduced in mice injected with ApoE-shRNA cells (2.7 ± 1.6) compared to mice injected with NC-shRNA cells (15.3 ± 4.1) (Supplementary Figure 4B). Also, histological analysis showed that lung metastatic tumor development was inhibited in the mice injected with ApoE-shRNA cells (Supplementary Figure 4C). These results supported that ApoE may contribute to tumor growth and metastasis.

### Activation of NK Cells and Cytotoxic CD8 T Cells at Tumor Sites of ApoE KO Mice

**Figure 4:**
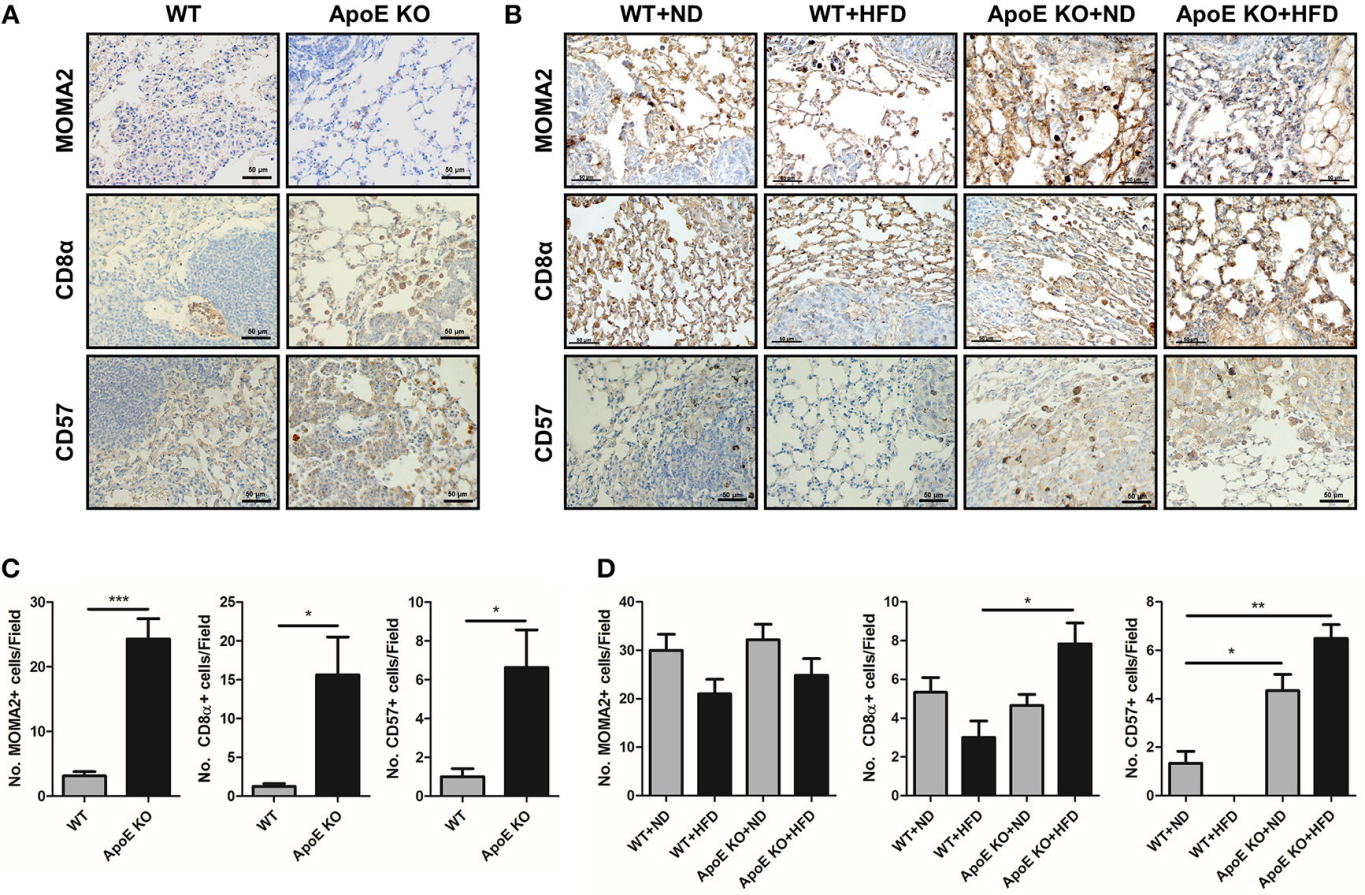
Effect of ApoE KO on the infiltration of immune cells into urethane-induced lung tumor tissues and B16F10 lung metastasis tissues. **(A,B)** Representative immunohistochemical images showing MOMA2 (for monocytes/macrophages), CD8α (for cytotoxic CD8 T cells) and CD57 (for NK cells) positive cells in the urethane-induced lung tumor tissues **(A)** and B16F10 lung metastasis tissues **(B)** from WT and ApoE KO mice. Scale bar, 50 μm. **(C,D)** Bar graphs represent infiltrated cells in tumor area. ^*^p < 0.05, ^**^p < 0.01, and ^***^p < 0.001.

Previously reports revealed that cytotoxic CD8α T cells and NK cells in ApoE KO mice were more cytotoxic compared with WT mice (22, 35). Thus, we investigated whether CD8α T cells and NK cells are involved in tumor growth inhibition in ApoE KO mice. Using immunohistochemistry, we detected several CD8α+ T cells and CD57+ NK cells at tumor sites in urethane-induced lung tumor tissues. Immunohistochemical staining of urethane-induced lung tumor tissues revealed that the number of CD8α+ T cells was 16.3 ± 13.6 cells/field in ApoE KO mice, but only 1.3 ± 1.6 cells/field in WT mice, and the number of CD57+ NK cells was 5.9 ± 6.4 cells/field in ApoE KO mice, but only 0.29 ± 0.49 cells/field in WT mice (Figures 4A,C). In lung metastasis tissues, CD8α+ T cells in HFD-fed ApoE KO mice (7.5 ± 2.61 cells/field) were more in number than in HFD-fed WT mice (3.0 ± 2.88 cells/field) (Figures 4B,D). Increased number of CD57+ NK cells were found infiltrated in HFD-fed ApoE KO mice (6.8 ± 1.3 cells/field) than in HFD-fed WT mice (0.17 ± 0.44 cells/field) (Figures 4B,D). This significant increase of cytotoxic cell number indicated that the inhibitory effect of ApoE KO on tumor growth and metastasis may be associated with increase of infiltration of CD8α+ T cells and CD57+ NK cells in urethane-induced lung tumor tissues and lung metastasis tissues.

### Effect on ApoE KO on NK Cell-Mediated Cytotoxicity

**Figure 5:**
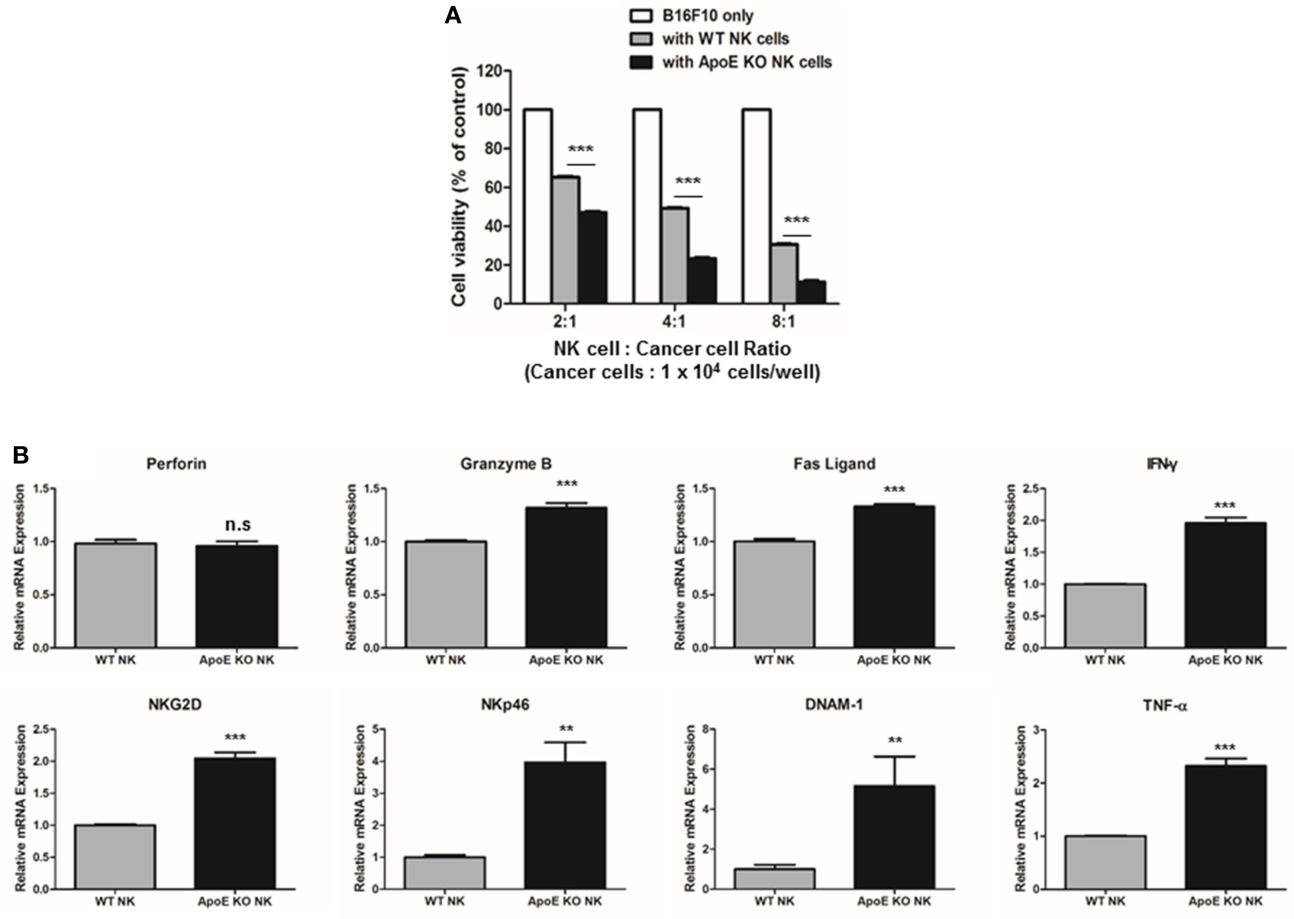
Effect on ApoE KO on NK cell-mediated cytotoxicity. **(A)** NK cell cytotoxicity was determined using LDH assay co-cultured with WT or ApoE KO NK cells and B16F10 target cells for 4 h (1 × 10^4^ cells/well). **(B)** Perforin, Granzyme B, Fas L, TNF-α, IFN-γ, NKG2D, NKp46, and DNAM-1 mRNA expressions in WT or ApoE KO NK cells were determined by qPCR (*n* = 3). ^**^p < 0.01 and ^***^p < 0.001.

Higher infiltration of CD8 T cells and NK cells in both urethane-induced lung tumor tissues and lung metastasis tissues was observed *in vivo*. Among these cells, NK cells are important for innate and adaptive immunity and play a critical role in tumor growth and metastasis (36). Therefore, we investigated whether ApoE expression affects the cytotoxic function of NK cells. Using co-culture of B16F10 mouse melanoma cells and spleen-derived NK cells from WT or ApoE mice, we found that NK cells derived ApoE mice (ApoE KO NK cells) showed more significantly increased cytotoxicity compared to NK cells derived from WT mice (WT NK cells) (Figure 5A). We studied the expression of perforin, granzyme B, TNF-α, IFN-γ, Fas L, NKG2D, NKp46, and DNAM-1, which are related with NK cell-mediated cytotoxicity, in NK cells from WT and ApoE KO mice. We found that expression of granzyme B, TNF-α, Fas L, IFN-γ, NKG2D, NKp46, and DNAM-1 in ApoE KO NK cells was higher (1.31-fold granzyme B, 2.32-fold TNF-α, 1.33-fold Fas L, 1.95-fold IFN-γ, 2.04-fold NKG2D, 3.46-fold NKp46, and 4.36-fold DNAM-1) compared to that in WT NK cells (Figure 5B). These results indicate that ApoE KO augmented NK cell-mediated cytotoxicity and these effects might due to the induction of granzyme B, TNF-α, Fas L, IFN-γ, NKG2D, NKp46, and DNAM-1 expression.

### TREM-1 Augments NK Cell-Mediated Cytotoxicity Through T-bet Expression

**Figure 6:**
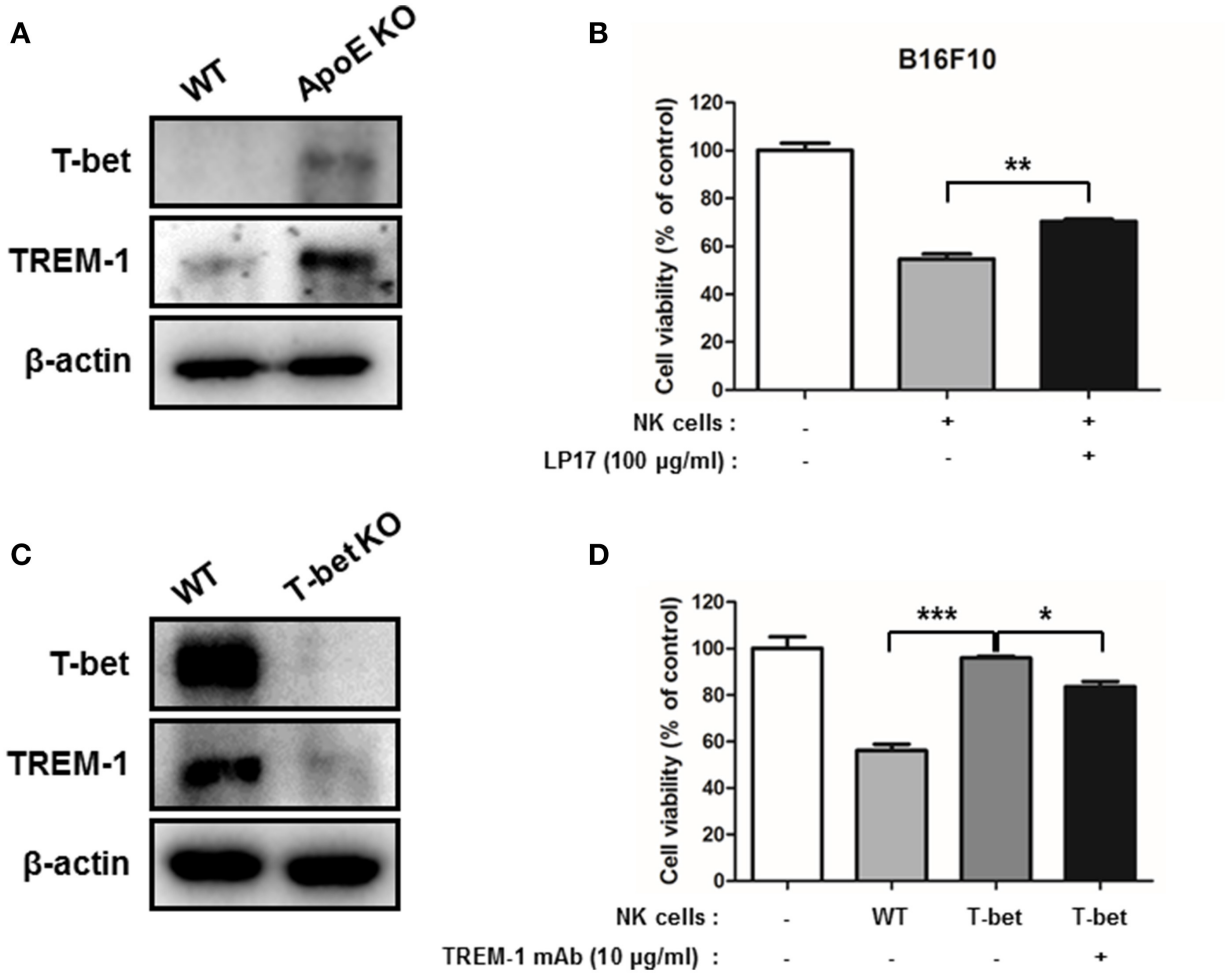
TREM-1 is involved in NK-cell mediated cytotoxicity. WT and ApoE KO NK **(A)** or T-bet KO NK **(C)** cell lysates were analyzed by Western blotting. NK cell cytotoxicity was determined using LDH assay co-cultured with control WT, LP-17 treated WT NK **(B)** or with control WT, non-treated or TREM-1 mAb treated T-bet KO NK cells **(D)** and B16F10 target cells for 4 h (1 × 10^4^ cells/well) (*n* = 3). E:T ratio is 4:1. ^*^p < 0.05, ^**^p < 0.01, and ^***^p < 0.001.

TREM-1 is expressed in NK cells and its expression is associated with lung cancer progression in patients (37, 38). Moreover, GWAS analysis demonstrated that ApoE is associated with TREM-1 (Supplementary Figure 5). To investigate the role of TREM-1 in lung cancer, we studied the expression of TREM-1 in urethane-induced lung tumor tissues and B16F10 lung metastasis tumor tissues using western blotting and immunohistochemistry. Western blot and immunohistochemistry analysis demonstrated that TREM-1 is up-regulated in ApoE KO mice in both urethane-induced lung tumor and B16F10 lung metastasis tumor tissues (Figures 1, 2 and Supplementary Figures S2A,B). TREM-1 is usually expressed in immune cells, NK cells, monocytes, and macrophages, and is associated with innate immune responses (24). In addition, a previous study showed that T-bet, also known as TBX21, could regulate TREM-1 expression in monocytes/macrophages (38). We therefore investigated the relationship between TREM-1, T-bet, and NK cell-mediated cytotoxicity. First, we compared the expression level of TREM-1 and its potential regulator T-bet in NK cells from WT mice and ApoE KO mice. We found that TREM-1 and T-bet expression was significantly higher in ApoE KO NK cells than in WT NK cells (Figure 6A). To identify whether TREM-1 was associated with NK cell-mediated cytotoxicity, WT NK cells were treated with LP-17, an antagonistic TREM-1 peptide. In co-culture experiments with B16F10 and WT NK cells, NK cell-mediated cytotoxicity was reduced when they were blocked with LP-17 (Figure 6B). A previous study revealed that T-bet may regulate TREM-1 expression in monocytes/macrophages (38). We investigated TREM-1 expression in NK cells from WT and T-bet KO mice. We found that NK cells from T-bet KO mice (T-bet KO NK cells) showed significantly decreased level of TREM-1 compared to those from WT mice (Figure 6C). In NK cell biology, T-bet plays a pivotal role in NK cell function, namely maintenance of IFN-γ, and is involved in cytotoxicity (39). However, the relationship between T-bet and TREM-1 in NK cell-mediated cytotoxicity is unknown. Therefore, we performed cytotoxicity assays using TREM-1 mAb-treated NK cells and non-treated NK cells from T-bet KO mice. NK cells derived from T-bet KO mice did not show cytotoxic effect against B16F10 melanoma. However, TREM-1 mAb-treated T-bet KO mice derived NK cells showed higher cytotoxicity than non-treated T-bet KO NK cells (Figure 6D). Thus, increased TREM-1 may enhance NK cell-mediated cytotoxicity in ApoE KO mice.

### Lung Tumor Tissue Microarray Analysis

**Figure 7:**
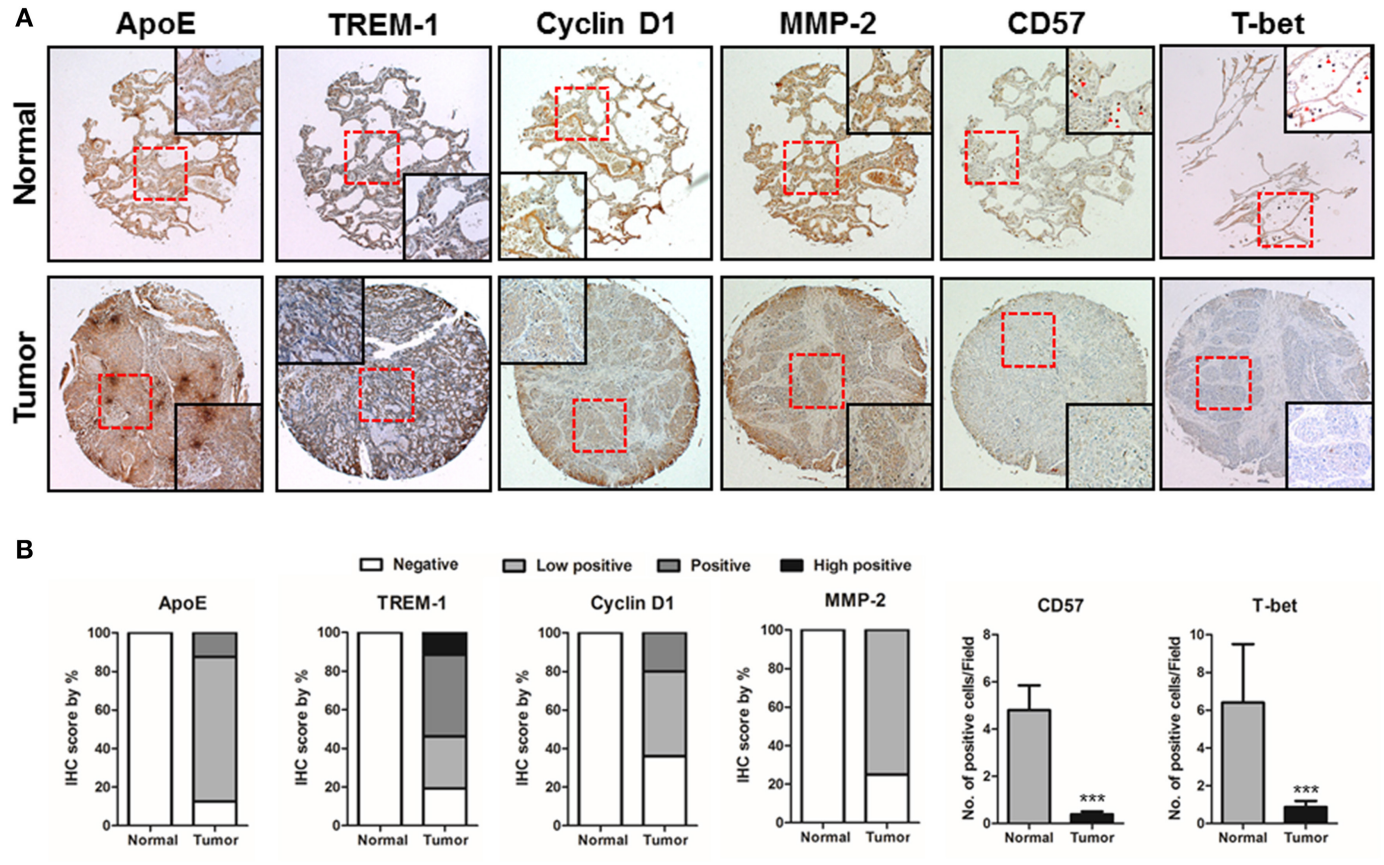
ApoE, TREM-1, Cyclin D1, MMP-2, CD57, and T-bet expressions in the progression of lung tumors. **(A)** Tissue microarray analysis showing the expression of ApoE, TREM-1, Cyclin D1, MMP-2, CD57, and T-bet during lung tumor progression in normal and tumor tissue samples. Representative immunohistochemical images of each groups. **(B)** Bar graphs showing the ratio of ApoE, TREM-1, Cyclin D1, MMP-2, CD57, and T-bet expressions. Tissue microarray samples are contained of 10 samples from normal lung tissues and 140 samples from lung tumor tissues. ^***^p < 0.001.

In this study, ApoE KO showed reduced lung tumor development but increased TREM-1 expression in ApoE KO lung tumor tissues. We analyzed whether ApoE and/or TREM-1 expression is associated with tumor development in lung tumor patients. We performed a tissue microarray screening using samples from lung tumor patients at 2 or 3 stages of tumor progression. We found that expression of ApoE and tumor growth-related proteins Cyclin D1 and MMP-2 was higher in the lung tumor patient tissues, but TREM-1 expression and the number of CD57 and T-bet positive cells were lower compared to normal tissues (Figures 7A,B). Additionally, we investigated the ApoE expression levels in normal and lung cancer patients. Oncomine analysis of okayama lung cancer database showed that expression of ApoE was upregulated in lung cancer **patients** (Supplementary Figure 6). Further analysis indicated that high ApoE expression exhibits a negative prognostic value for 5-year survival and recurrence (Supplementary Figure 6).

## Discussion

In this study, we found that carcinogen-induced lung tumor development and B16F10 lung metastasis were inhibited in ApoE KO mice in comparison with WT mice. The inhibitory effects of ApoE KO on tumor growth and metastasis were associated with an increased number of infiltrated immune cells, CD57+ NK cells, and TREM-1 expression at tumor tissues. We also found that inhibitory effects on lung tumor growth in ApoE KO mice was associated with TREM-1-mediated NK cell cytotoxicity.

ApoE is an important lipoprotein that mediates cholesterol transport and metabolism (12). Abnormal function of ApoE is mainly associated with Alzheimer's disease, atherosclerosis, and cardiovascular disease (40, 41). However, recent studies have revealed that ApoE is associated with cancer progression and metastasis. ApoE KO mice showed increased tumor development and pulmonary metastases in a mammary cancer cell orthograft model and in B16F10 mouse melanoma metastasis in comparison to WT mice (18, 19). Contradictory results have also been reported. Knockdown of ApoE in ovarian cancer cells induced cell cycle arrest and apoptosis (13). Suppression of ApoE in lung adenocarcinoma cells reduced colony formation, chemotherapy resistance, cell proliferation, and metastasis (16). It is not currently clear how these different results can be explained. However, the explanation is probably not related to the control of cholesterol metabolism by ApoE, because our results showed that cholesterol levels were not associated with tumor growth in ApoE KO mice. Moreover, our *in vitro* study also indicates that the level of cholesterol did not alter cancer cell growth. These data indicate that the inhibitory effect on tumor growth may not be associated with cholesterol level. We thus assume that ApoE KO iiself may be significant for cancer cell growth.

Thus, it is noteworthy that ApoE knockout and knockdown led to decreased expression of cyclin D1 and its associated proteins CDK4 and CDK6, in urethane-induced lung tumors, lung metastases, and cancer cell lines. Expression of metastasis-associated proteins MMP-2 and MMP-9 was also decreased when ApoE was suppressed. In agreement with our data, low-density lipoprotein receptor-related protein-1 (LRP-1) and ApoE-binding receptors promote cancer cell migration via induction of MMP-2 and MMP-9 expression, and are involved in inducing cyclin D1 in interstitial fibroblasts (42, 43). Clinical analyses showed that ApoE expression was increased in breast, ovarian, gastric, prostate and lung cancers (44–48). Serum ApoE levels were elevated in NSCLC patients compared with normal controls, and were associated with tumor stages (15). Another study also showed that increased ApoE expression was clinically significantly correlated with malignant pleural effusion-associated lung cancer patient survival (16). These results were in accord with those of our tissue microarray analysis and Oncomine analysis that ApoE expression was elevated in lung cancer patients. Taken together, our results suggest that ApoE itself may control tumor signaling and promote tumor development and metastasis, rather than control tumor growth through cholesterol metabolism. However, the underlying molecular mechanisms are not clear.

NK cells and Tc cells are main effectors in the immune response against viral infection and tumors (49). Organ-associated NK cells and elevated IFN-γ production by NK cells inhibited experimental metastasis in the lung (50). It was also reported that significantly decreased expression of perforin, granzyme B, and IFN-γ in T, NKT-like, and NK cells is associated with lung cancer tissues compared with normal lung tissues from patients (51). The number of tumor-infiltrating NK cells is associated with survival of squamous cell lung cancer patients (52). These facts accord with our results revealing that ApoE KO mice showed more infiltrated CD57+ NK cells and CD8α+ T cells at tumor tissues compared to WT mice. *In vitro*, NK cells from ApoE KO mice were more cytotoxic than those from WT mice in co-culture studies with NK cells and cancer cells. Furthermore, NK cells derived from ApoE KO mice showed increased expression of Granzyme B, TNF-α, Fas L, IFN-γ, and NKG2D, which could regulate NK cell-mediated cytotoxicity. In this regard, it is noteworthy that ApoE could be related to lymphocyte recruitment. ApoE KO mice showed increased NK cells infiltrated into atherosclerotic lesions, and increased release of IFN-γ (35, 53). Among the immune cells, ApoE is not expressed on NK cells. Also, we tried to confirm the expression of ApoE in NK cells, but we cannot detect using western blot analysis (data not shown). Although further study needs the relationship between NK cell differentiation or maturation and ApoE, we thought that NK cells may change more aggressive phenotype during differentiation or maturation in ApoE KO mice. There is a little clue about relationship between ApoE and antitumor activity. Previous report showed that the mRNA expression or serum level of Th1 cytokines, such as IFN-γ, IL-1β, IL-2, and IL-6, and intercellular adhesion molecule 1 (ICAM-1) were increased, but IL-4 was decreased in liver of ApoE KO mice compared to WT mice (54). Previous reports indicated that IL-2 activates NK cells and IL-4 could mediate downregulation of NKG2D (55). Also, ICAM-1 is involved to promote increased target cell death (56). Thus, NK cells could be more cytotoxic in ApoE KO condition.

TREM-1 is known to be an immune regulator against viral infection, septic shock, pneumonia, and asthma (24). However, in cancer biology, the role of TREM-1 is largely unknown. In lung cancer patients, TREM-1 expression is upregulated in tumor-associated macrophages and is correlated with clinical outcome (28, 37). However, a recent study showed that expression levels of TREM-1 in tumor associated macrophages and blood monocytes were significantly decreased during lung tumor progression (38). TREM-1 could be also linked with polymorphonuclear cell-mediated cytotoxicity (57). Following these previous studies, we hypothesized that an increase of NK cell-mediated anti-tumor effect due to ApoE KO could be involved with TREM-1 expression. To identify whether TREM-1 is involved in NK cell-mediated cytotoxicity, we used LP-17, a TREM-1 antagonist. We found that blockade of TREM-1 reduced NK cell mediated cytotoxicity. Thus, TREM-1 is positively correlated with anti-tumor function of NK cells. T-bet is critical associated with TREM-1 expression, and it expressed in various immune cells (39, 58). In NK cell function, T-bet is involved in NK cell cytotoxicity via the regulation of IFN-γ, perforin, and granzyme B releases (39, 59). In addition, it was reported that T-bet KO mice showed lower expression of TREM-1 in monocytes/macrophages compared to WT mice (38). In this study, we found that NK cells derived from T-bet KO mice showed a lower level of TREM-1 than those from WT mice. NK cells derived from T-bet KO mice showed significantly reduced cytotoxicity compared with those from WT mice. However, TREM-1 mAb recovered cytotoxic ability of NK cells derived from T-bet KO mice. Furthermore, gene network analysis revealed that ApoE, TREM-1, and T-bet were connected via DNAX activation protein of 12 kDa (DAP12), known as TYROBP, which is coupled with TREM-1. These results implied that ApoE deficiency enhanced NK cell-mediated anti-tumor effect via an increase of T-bet-dependent TREM-1 expression. In this regard, we found increased expression of TREM-1 and T-bet in NK cells derived from ApoE KO mice compared to those from WT mice. Moreover, we found elevated expression of TREM-1, and increased CD57 and T-bet positive NK cells in lung tumor patient tissues. These data indicate that TREM-1 could be associated with ApoE KO-mediated suppression of tumor growth through increased anti-tumor activity of NK cells.

## Ethics Statement

This study was carried out in accordance with the recommendations of Chungbuk National University Animal Care Committee. The protocol was approved by the Chungbuk National University Animal Care Committee (CBNUA-929-16-01).

## Author Contributions

YL conducted most of the experiments, performed data analysis, generated most of the experimental mice and was the primary writer of the manuscript. IY, KK, and S-BH provided advice throughout the project. JH supervised the entire project and had a major role in experimental design, data interpretation, and writing the manuscript.

### Conflict of Interest Statement

The authors declare that the research was conducted in the absence of any commercial or financial relationships that could be construed as a potential conflict of interest.
