# Corrigendum: Inhibition of Lung Tumor Development in ApoE Knockout Mice *via* Enhancement of TREM-1 Dependent NK Cell Cytotoxicity

**DOI:** 10.3389/fimmu.2021.840856

**Published:** 2022-01-21

**Authors:** Yong Sun Lee, In Jun Yeo, Ki Cheon Kim, Sang-Bae Han, Jin Tae Hong

**Affiliations:** College of Pharmacy and Medical Research Center, Chungbuk National University, Cheongju, South Korea

**Keywords:** Lung tumor development, apolipoprotein E, TREM-1, T-bet, NK cells

In the original article, there was a mistake in **Figure 3C** as published. In **Figure 3C** **,** overlapped images were mistakenly used. For reliability, the experiment (transwell migration assay) was performed again, and the description of the experimental method was revised. The corrected **Figure 3** with a legend and a revised method appear below.

**Figure 3:**
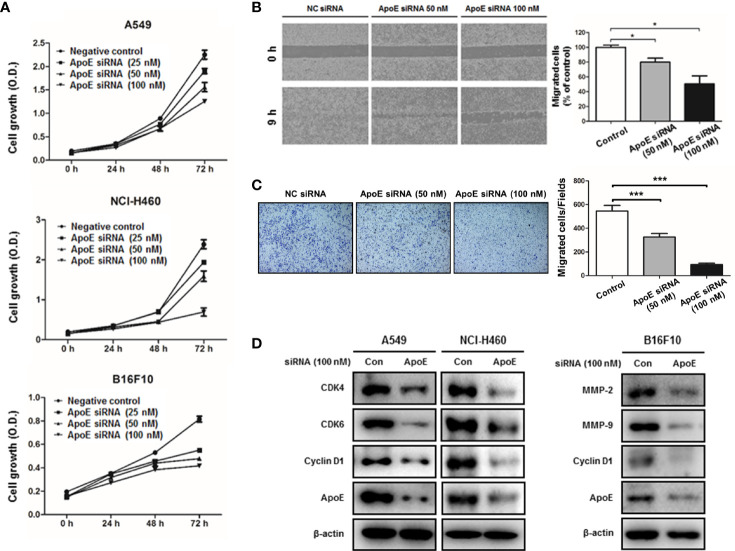
Effect of ApoE knockdown on cell growth and migration in cancer cells. **(A)** Lung cancer cells (A549 and NCI-H460) and B16F10 cells were plated on 96-well plates (1x10^3^ cells per well) and transfected with the negative control (NC) siRNA or ApoE siRNA (25, 50 or 100 nM) for indicated time points. Cell growth was measured by MTT assay (*n*=5). **(B)** B16F10 cells were seeded on μ-Slide and transfected with NC siRNA or ApoE siRNA (50 or 100 nM). Cells were cultured to confluent on μ-Slide (*n*=3). After silicon-wall removal, cells were allowed to migrate into cell-free zone. Cell migration was detected at various times post-silicon-wall removal by a microscopy at 100X. **(C)** B16F10 cells transfected with NC siRNA or ApoE siRNA (50 or 100 nM) were seeded onto transwell inserts pre-coated with collagen on the bottom side and loaded into culture well filled with growth medium containing 10% FBS as a chemoattractant (*n*=3). After 18 h incubation, transwell inserts were fixed and stained by crystal violet solution. Bar graphs represent cell-migration distance or number of migrated cells. *p < 0.05 and ***p < 0.0001. **(D)** Cells were transfected with NC siRNA or ApoE siRNA (100 nM) for 24 h. Cell extracts were analyzed by Western blotting. Samples (30 μg) were resolved on SDS–PAGE and detected with specific antibodies against CDK4, CDK6, Cyclin D1, MMP-2, MMP-9 and ApoE. β-actin was used as a loading control.

The authors apologize for this error and state that this does not change the scientific conclusions of the article in any way. The original article has been updated.

## Transwell migration assay

Serum-starved B16F10 cells (1 × 10^5^ cells in 100 μl serum-free medium) were added to the collagen pre-coated transwell insert (pore size 8.0 μm; distance 6.5 mm), and the culture well was filled with 600 μl of the complete medium containing 10% FBS. After 18 h, cells were fixed with 70% ethanol for 10 min and stained with crystal violet solution. Then, non-migrated cells in the upper side of transwell insert were removed using a cotton swab. Three random fields of each insert were counted and photographed under a light microscope (x100, Olympus, Tokyo, Japan).

## Conflict of Interest

The authors declare that the research was conducted in the absence of any commercial or financial relationships that could be construed as a potential conflict of interest.

## Publisher’s Note

All claims expressed in this article are solely those of the authors and do not necessarily represent those of their affiliated organizations, or those of the publisher, the editors and the reviewers. Any product that may be evaluated in this article, or claim that may be made by its manufacturer, is not guaranteed or endorsed by the publisher.

