# Supplementary material for: Inhibition of Lung Tumor Development in ApoE Knockout Mice via Enhancement of TREM-1 Dependent NK Cell Cytotoxicity
Source: Front Immunol. 2019 Jun 18;10:1379. doi: 10.3389/fimmu.2019.01379 (PMC6592261; doi:10.3389/fimmu.2019.01379)
Supplement: Supplementary file 1 [file Presentation_1.pdf]

Supplementary Table S1. Primary antibody information

Name	Cat. No.	Application	Supplier
PCNA (C-20)	sc-9857	WB, IHC	Santa Cruz Bio
CDK4 (DCS-35)	sc-23896	WB	Santa Cruz Bio
CDK6 (B-10)	sc-7961	WB	Santa Cruz Bio
Cyclin D1 (DCS-6)	sc-20044	WB, IHC	Santa Cruz Bio
MMP-2 (8B4)	sc-13595	WB	Santa Cruz Bio
MMP-9 (E-11)	sc-393859	WB	Santa Cruz Bio
Bcl-2 (C-2)	sc-7382	WB	Santa Cruz Bio
p-JNK (G-7)	sc-6254	WB	Santa Cruz Bio
JNK (FL)	sc-571	WB	Santa Cruz Bio
p-ERK1/2 (Thr 202)	sc-101760	WB	Santa Cruz Bio
p-STAT3 (B-7)	sc-8059	WB	Santa Cruz Bio
STAT3 (F-2)	sc-8019	WB	Santa Cruz Bio
CD8α (D-9)	sc-7970	IHC	Santa Cruz Bio
β-actin (C4)	sc-47778	WB	Santa Cruz Bio
TREM-1	R30663	WB, IHC	NSJ Bio
TREM-1	bs-10306R	IHC	Bioss antibodies
T-bet	bs-3599R	WB, IHC	Bioss antibodies
Caspase-3	9662S	WB	Cell signaling Technology
ERK	9102S	WB	Cell signaling Technology
p-p38 (T180/Y182)	4511S	WB	Cell signaling Technology
p38 (D13E1)	8690S	WB	Cell signaling Technology
ApoE	ab1907	WB, IHC	Abcam
MOMA2	ab33451	IHC	Abcam
CD57	ab199156	IHC	Abcam
MMP-2	ab92536	IHC	Abcam

Supplementary Table S2. List and sequences of qPCR primers for mRNA expression

Primer		Sequences
18s	Sense (5'-3')	AGGAATTGACGGAAGGGCACCA
	Anti-sense (5'-3')	GTGCAGCCCCGGACATCTAAG
Perforin	Sense (5'-3')	CGTGAGCGTCACGTCGAA
	Anti-sense (5'-3')	GTTCCCGAAGAGCAGATCATG
Granzyme B	Sense (5'-3')	CCACTCTCGACCCTACATGG
	Anti-sense (5'-3')	GGCCCCCAAAGTGACATTTATT
Fas Ligand	Sense (5'-3')	TCCGTGAGTTCACCAACCAAA
	Anti-sense (5'-3')	GGGGGTTCCCTGTTAAATGGG
TNF-α	Sense (5'-3')	TCTTCTCATTCCTGCTTGTGG
	Anti-sense (5'-3')	CACTTGGTGGTTTGCTACGA
NKG2D	Sense (5'-3')	CGATTCACCCTTAACACATTGATG
	Anti-sense (5'-3')	GGGACTTCCTTGTTGCACAATAC
IFN-γ	Sense (5'-3')	GCCATCAGCAACAACATAAGCGTC
	Anti-sense (5'-3')	CCACTCGGATGAGCTCATTGAATG
NKp46	Sense (5'-3')	ATGCTGCCAACACTCACTG
	Anti-sense (5'-3')	GATGTTCACCGAGTTTCCATTTG
DNAM-1	Sense (5'-3')	TCGCTCAGAGGCCATTACAG
	Anti-sense (5'-3')	CCCTGGGCTCTTTAAGTGGAA

(A)

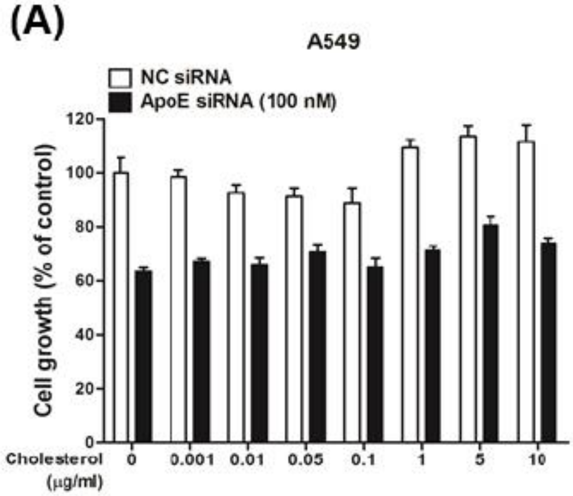

(B)

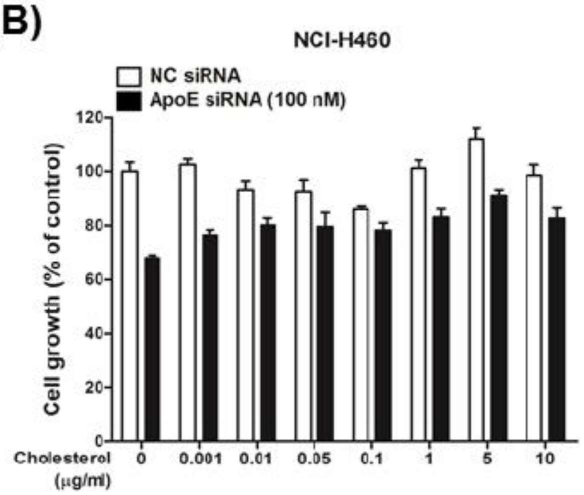

(C)

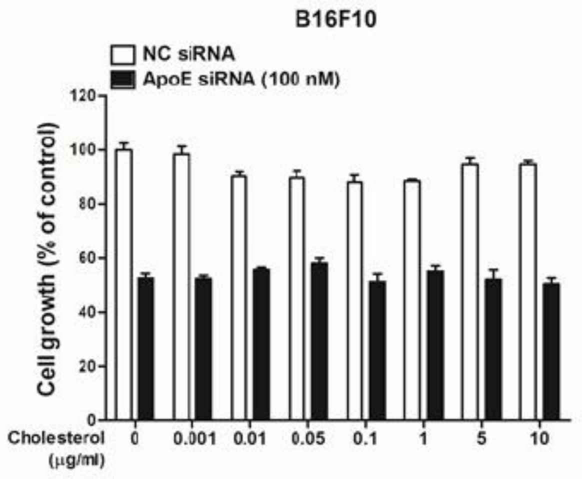

Supplementary Figure S1. Effect of cholesterol on cancer cell viability. (A-C) Lung cancer cells (A549 and NCI-H460) and B16F10 cells were plated on 96-well plates **($5x10^3$** cells per well) and transfected with NC siRNA or 100 nM ApoE siRNA for 24 h (*n=5*). Cells were then treated with cholesterol (indicated concentrations) for another 24 h. Cell viability was measured by MTT assay.

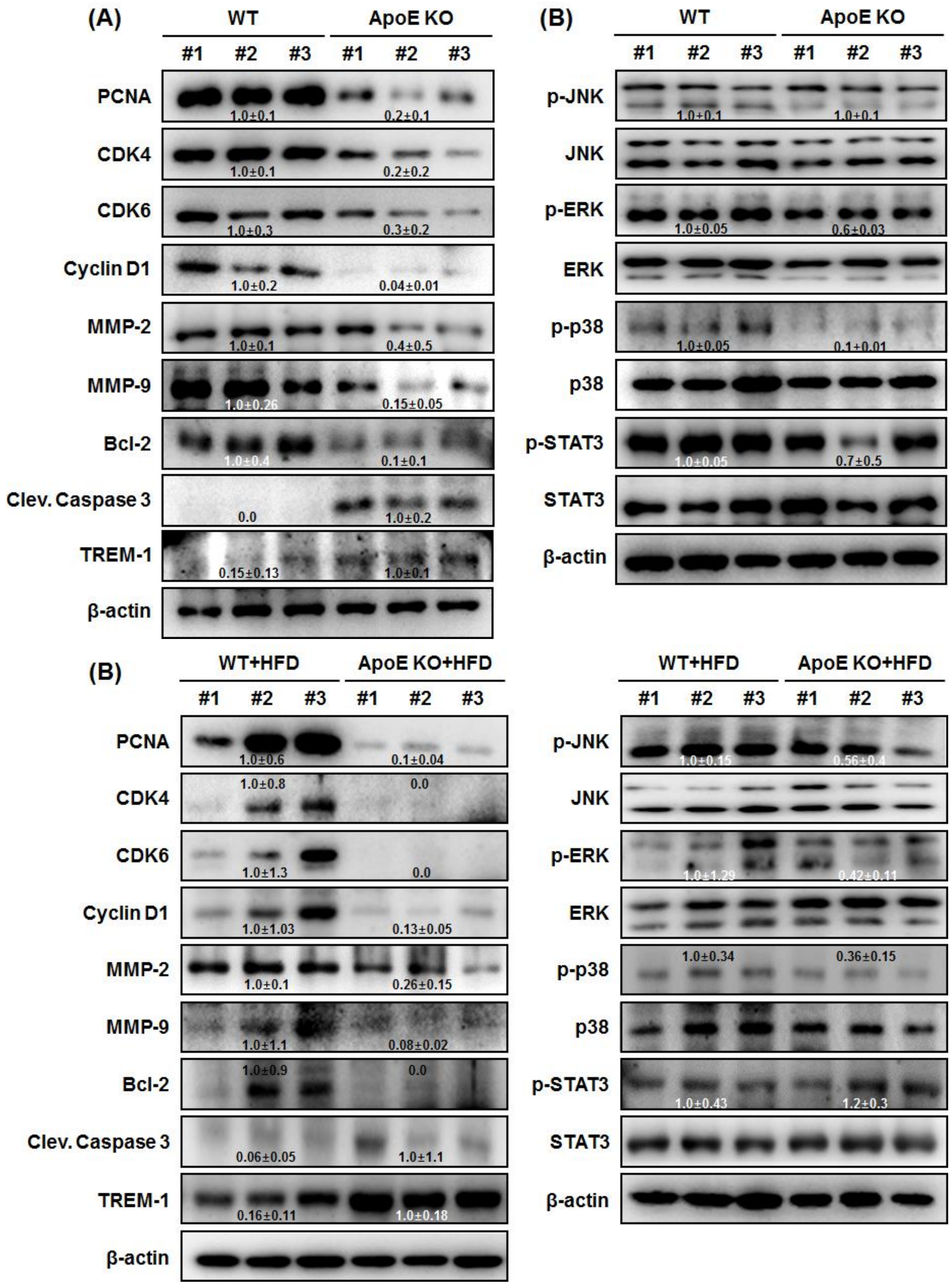

Supplementary Figure S2. Effect of ApoE knockout on tumor development or metastasis related protein expression in urethane induced lung tumor tissues and B16F10 lung metastasis tissues. (A-B) Urethane-induced or B16F10 lung metastasis tumor extracts were analyzed by Western blotting. Samples (30 μg) were resolved on SDS–PAGE and detected with antibodies against PCNA, CDK4, CDK6, Cyclin D1, MMP-2, MMP-9, Bcl-2, cleaved caspase-3, TREM-1, p-JNK, JNK, p-ERK, ERK, p-p38, p38, p-STAT3 and STAT3. β-actin was used as a loading control.

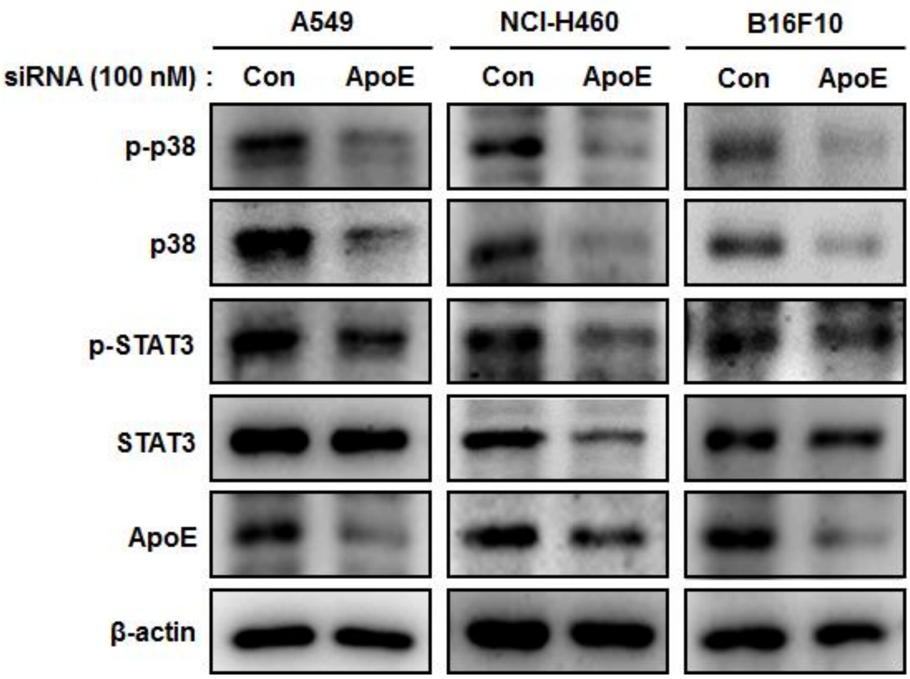

Supplementary Figure S3. Effect of ApoE knockdown on tumor development and metastasis related protein expression in cancer cells. Lung cancer cells (A549 and NCI-H460) or B16F10 cells were transfected with NC siRNA or ApoE siRNA (100 nM) for 24 h. Cell extracts were analyzed by Western blotting. Samples (30 μg) were resolved on SDS–PAGE and detected with antibodies against p-p38, p38, p-STAT3, STAT3 and ApoE. β-actin was used as a loading control.

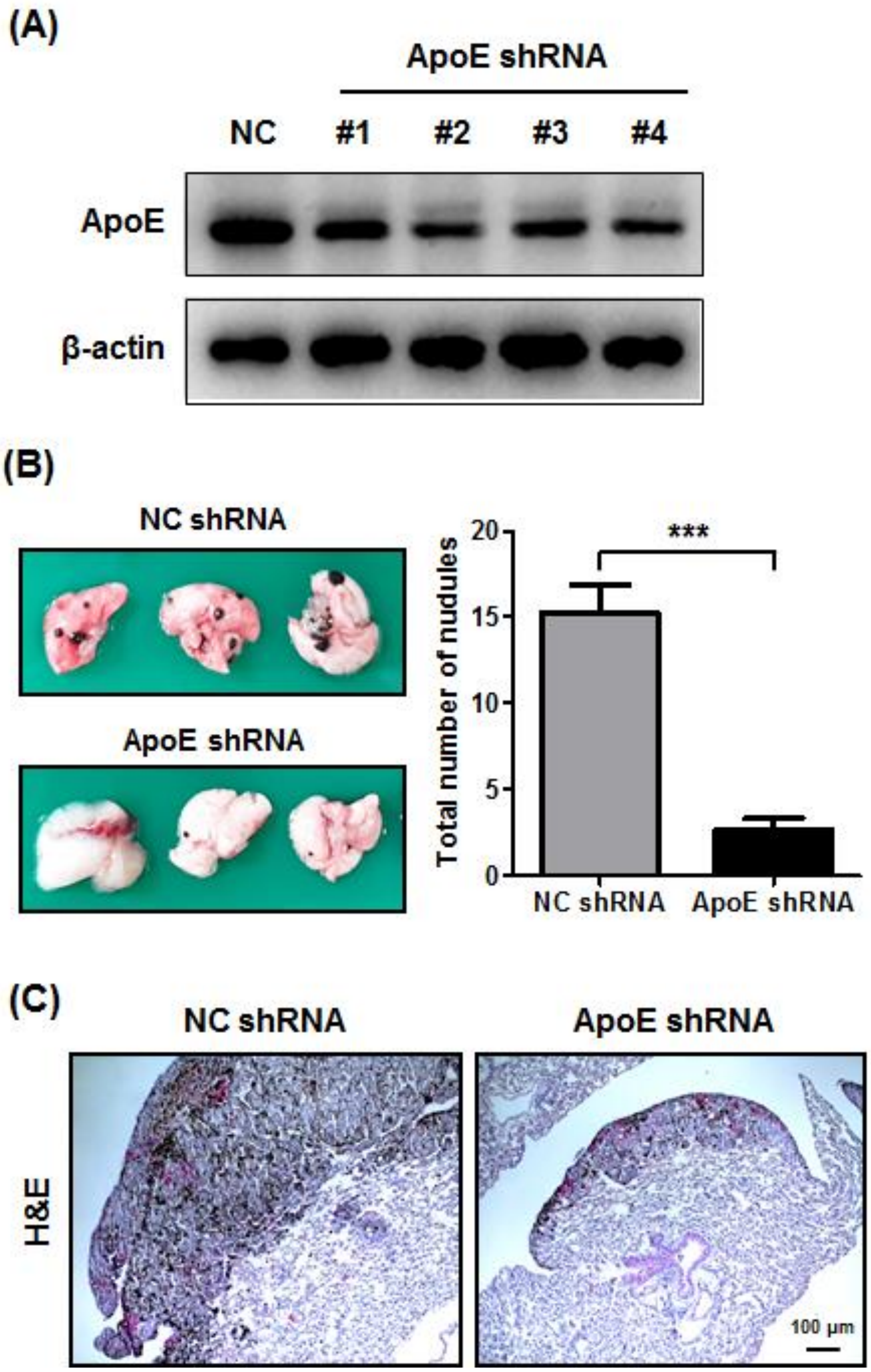

Supplementary Figure S4. Effect of ApoE knockdown B16F10 cells on lung metastasis. (A) B16F10 cells were transfected with NC or ApoE shRNA. ApoE-shRNA cells were selected by Puromycin treatment for 3 weeks. ApoE expression was confirmed by Western blotting. (B) NC or ApoE-shRNA cells were intravenously injected at mouse tail vein ($4x10^4$ cells/mouse) (n=7). After 21 days, mice were sacrificed, and lung metastatic nodules were visualized and counted. (C) Lung metastasis tissues were stained with haematoxylin and eosin. Scale bar, 100 μm. ***p<0.001.

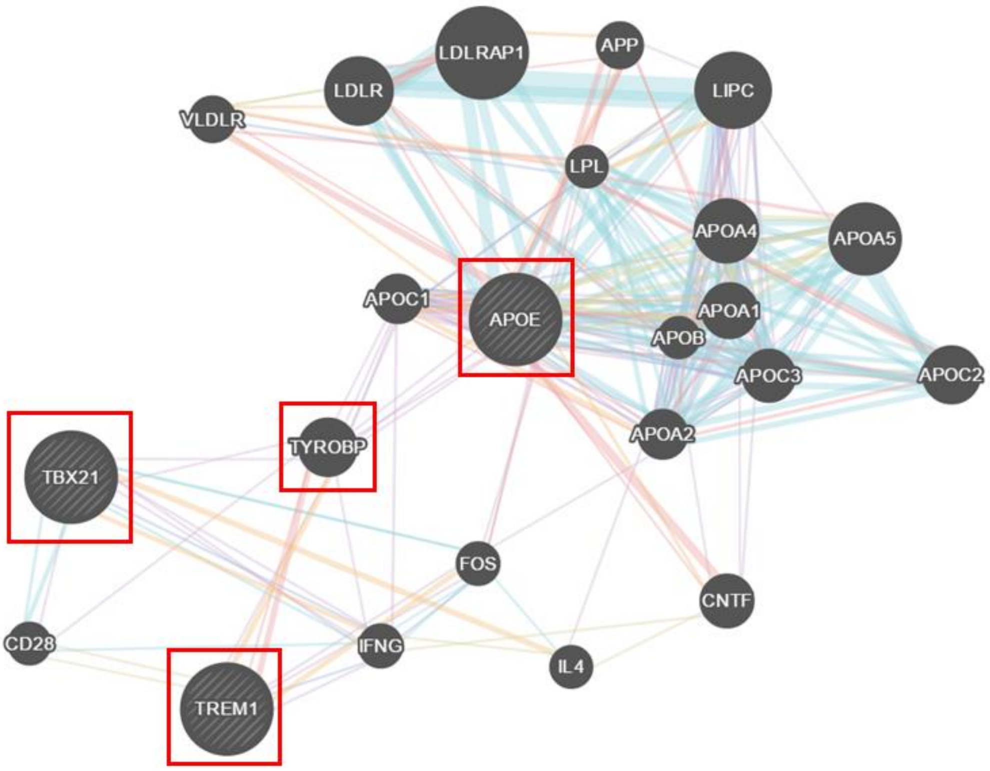

Supplementary Figure S5. Gene network analysis using GeneMANIA. The gene map of ApoE, TREM-1 and T-bet (also known as TBX21) is shown based on known functional association networks.

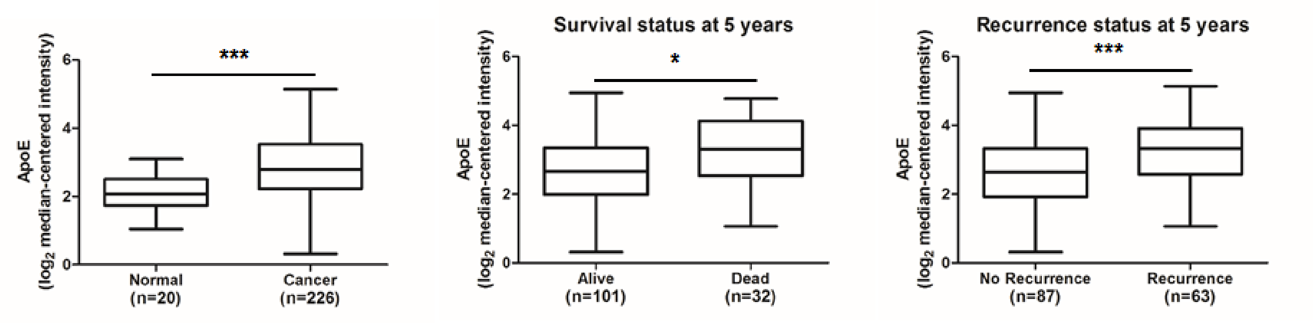

Supplementary Figure S6. Expression of ApoE in lung cancer patients. ApoE expression from Oncomine analysis of the okayama lung cancer database. Values are normalized to $\log_2$ median-centered intensity. ***p<0.05 and ***p<0.001.**
